# Visualization of the renal artery in kidney transplant patients using time-resolved computed tomography angiography

**DOI:** 10.1177/20584601211046334

**Published:** 2021-10-01

**Authors:** Svensson-Marcial Anders, Genberg Helena, Brehmer Katharina, Themudo Raquel, Brismar B Torkel

**Affiliations:** 1 Department of Radiology, 27106 Karolinska University Hospital, Huddinge, Sweden; 2 Division of Medical Imaging and Technology, Department of Clinical Science, 27106 Intervention and Technology at Karolinska Institute, Stockholm, Sweden; 3 Department of Transplantation Surgery, 27106 Karolinska University Hospital, Huddinge, Sweden; 4 Departments of Molecular Medicine and Surgery, 27106 Clinical Physiology at Karolinska Institute, Stockholm, Sweden

**Keywords:** Computed tomography, time-resolved perfusion computed tomography angiography, transplant kidney, transplant renal artery stenosis, contrast agents-intravenous

## Abstract

**Background:**

Transplant renal artery stenosis (TRAS) is a post-operative complication which most often occurs between 3 months and 2 years after transplantation. TRAS is associated with kidney failure and hypertension and, thereby, with an increased risk of cardiovascular events.

**Purpose:**

The purpose of this retrospective study was to report our experience of perfusion computed tomography angiography (P-CTA) to identify a 50% lumen reduction (as compared to digital subtraction angiography, DSA), assess its subjective image quality and evaluate if contrast-induced acute kidney injury (CI-AKI) occurred.

**Material and Methods:**

All 13 patients who had undergone P-CTA for suspected TRAS at our institution were retrospectively evaluated. At P-CTA, eight or 12 g of iodine were administered intravenously, and five to seven scan sequences were merged into time-resolved images after motion correction. Eight patients underwent subsequent DSA.

**Results:**

The average patient weight was 76 kg (range 55–97 kg). Image quality was rated as good or excellent for all patients, and pathological changes were shown in 10 of 13 patients undergoing P-CTA. Two patients had a serum creatinine increase of >26 μmol/L during the first 3 days, but serum creatinine was significantly lower in all patients 1 month after P-CTA (165+/−69 μmol/L versus 232+/−66 μmol/L, *P* < .01). The diagnosis at P-CTA was verified in all eight patients who underwent DSA. However, in two cases with suspected stenosis, renal function was restored without angioplasty.

**Conclusion:**

Anatomy and blood flow of the transplant renal artery can be visualized using less than a third of the standard contrast media dose by using P-CTA technique.

## Introduction

Transplant renal artery stenosis (TRAS) is a post-operative complication which most often occurs between 3 months and 2 years after transplantation. TRAS is associated with kidney failure and hypertension and, thereby, with an increased risk of cardiovascular events. The reported risk of TRAS varies among different studies, ranging from 1% to 23% in all kidney transplantations.^1–3^ Early detection of TRAS is important to prompt adequate treatment and to preserve renal function. In clinical practice, TRAS is diagnosed primarily by Doppler ultrasound (DUS). The reference test for the definitive diagnosis of arterial stenosis is digital subtraction invasive angiography (DSA).^4,5^ Alternative non-invasive techniques for diagnosing TRAS are magnetic resonance angiography (MRA) and intravenous contrast media (CM)–enhanced computed tomography angiography (CTA).^6,7^ However, the nephrotoxicity of intravenous iodine contrast media (CM) is still a challenge to CM-enhanced imaging. Gadolinium-enhanced MRA is also contraindicated in patients with severe renal failure due to the risk of nephrogenic systemic fibrosis (NSF).^8,9^

The rapid evolution of CT technology has enabled the development of perfusion computed tomography angiography (P-CTA), by which multiple passages/sequences over the organ of interest are acquired. The technique can also be used for minimizing the CM dose at CT angiography (CTA).^10–13^ The high temporal resolution guarantees that a short CM bolus will be detected with optimal arterial enhancement. Using post-processing of image data, the rendered images can be shown as a movie, and the inflow of the CM in the vessels can be visualized as high-resolution time-resolved images.

The purpose of this retrospective study was to report our experience of P-CTA to identify a 50% lumen reduction (as compared to DSA), assess its subjective image quality, and evaluate if contrast-induced acute kidney injury (CI-AKI) occurred.

## Material and methods

This retrospective study was approved by the Regional Ethics Board, and informed consent was waived. All methods used in this study were carried out in accordance with local clinical radiology guidelines. The study complies with the Declaration of Helsinki. The inclusion criteria were a) patients in whom DUS had difficulty visualizing the renal artery and b) referred for P-CTA examination of the transplant kidney renal artery between April 2012 and Feb 2019. In total, 13 patients with clinical signs of TRAS were retrospectively included in the study. There were seven men and six women, aged 26–67 (mean 50) years and the time between kidney transplantation and P-CTA ranged between 12 and 270 days. All 13 patients had acute onset of hypertension and reduced kidney function (Table 1).

**Table 1.** Patient characteristics, estimated glomerular filtration rate per minute (eGFR, mL/min 1.73 m^2^) at the day of P-CTA, 3 days after and 30 days after CT. The amount of intravenous contrast media administered is stated in grams. The diagnosis (dx) at P-CTA and the number of days after the P-CTA that the DSA was performed and what interventions performed is also written out.

Pat. No	Age	Body weight (kg)	Body height (cm)	BMI (kg/m2)	Day of the P-CTA		Iodine given (g)	3 days after P-CTA		30 days after P-CTA		P-CTA dx	Day of DSA	Treatment at DSA
					eGFR	sCr		eGFR	sCr	eGFR	sCr			
1	46	56	153	24	34	155	12	56	104	53	108	Stenosis		
2	62	97	178	31	22	257	12	22	260	34	242	Stenosis	22	Ballon
3	46	78	185	23	21	294	12	33	203	37	185	Stenosis	53	Ballon
4	36	82	182	25	28	245	12	31	226	45	168	None	13	None
5	26	60	156	25	20	263	10	14	348	80	84	Stenosis	2	Stent
6	52	65	167	23	21	230	10	16	285	29	175	Not significant	14	None
7	54	91	191	25	32	204	10	29	217	51	137	None		
8	55	61	165	22	36	141	10	28	173	45	118	Stenosis		
9	59	93	173	31	28	218	12	53	127	56	121	Stenosis	1	Ballon
10	63	92	173	31	14	365	12	15	350	15	350	Kinked artery	62	Stent
11	36	71	154	30	14	307	8	25	215	52	118	None		
12	53	90	183	27	42	161	12	38	174	43	169	Stenosis	6	Ballon
13	67	55	160	21	26	173	10	29	160	27	170	Atero-sklerosis		

Notes: Patient: No.1 received anti-rejection therapy, No. 4 had angiotensin II-receptor blocker discontinued, No. 5 had DSA 2 days after P-CTA, No. 7 underwent P-CTA within 12 days from transplantation and had a recovery of transplantation-induced acute tubular necrosis in the early post-operative period, No. 9 had DSA the day after P-CTA, and No. 11 had a biopsy bleeding and subcapsular hematoma. This patient underwent surgical decompression.

All patients were scanned with a dual source 2 x 64 detector row MDCT (Siemens Somatom Definition Flash, Forchheim, Germany). The position of the transplant kidney was defined by non-CM scanning over the pelvis using automatic dose modulation, 100 kVp and 45 reference mAs. During P-CTA, a total volume of 20–30 mL (8–12 g (g) iodine) of iomeprol 400 mg I/mL, (Iomeron-400, Bracco Imaging SpA, Milan, Italy) was administered at 6 mL per second, followed by a saline chaser with the same injection flow through a 16-gauge peripheral venous access inserted in the antecubital vein. A fixed dose of 12 g of iodine was administered to the first four patients. From patient five and onward, the dosage was adjusted through visual assessment according to the patient’s body constitution. The scan delay was set to 15 s after the start of the CM injection. The P-CTA consisted of 5–7 scan repetitions over a range of 15 cm covering the transplant kidney, using 100 kVp and 70 mAs, giving a radiation dose below <9 millisievert (mSv).^14^ The CM enhancement of the iliac and renal arteries was monitored visually while scanning the different series, and the scan was manually ended when the enhancement started to decrease. Time resolution between series was 1.5 s.

All reconstructed series were sent to a dedicated post-processing workstation for motion correction and creation of a time-resolved merged data set (Syngovia, Siemens Healthcare, Forchheim, Germany). All multiplanar reformats (MPR), 3D volume rendering (VR), and 4D post-processing were carried out at a separate workstation (Advantage workstation 4.6, GE Healthcare, Milwaukee, WI, USA). The total post-processing time, including image transfer time between the CT and SyngoVia workstation, image motion correction, 3D reconstruction, 4D movie and image reformates, was approximately 45 min. With regard to retrospective analysis, two radiologists with 8 and 14 years of experience rated image quality on a five-grade scale (absent, poor, adequate, good, and excellent). The five-degree assessment of image quality summarized the visualization of the renal arteries, vascular anastomosis, and iliac arteries. The transplant renal artery was evaluated for the absence or the presence of luminal diameter reduction. From the thin axial slices, multiplanar reconstructions (MPR) were performed in order to visualize the artery in its long axis and in cross-sectional section perpendicular to the vessel long axis. When luminal reduction was present, the stenosis was graded as above or below 50 per cent luminal diameter narrowing.^15,16^ Luminal stenosis was assessed by visual assessment. Both reviewers were blinded to the DSA results. Reader disagreement was solved in consensus. Eight patients underwent DSA after the P-CTA. In six patients, DSA was performed within 1 month from P-CTA (1, 2, 6, 13, 14, and 22 days after P-CTA, respectively) and in two patients 53 and 62 days after P-CTA. The consensus from the P-CTA was compared with the findings reported by the interventionist at DSA.

Kidney function was evaluated by measurement of serum creatinine (sCr) on the day of the P-CTA examination, 3 days after and approximately >30 days after the P-CTA examination by calculating the estimated glomerular filtration rate (eGFR) using the Lund-Malmö formula**.** Student’s paired t-test, without Bonferroni correction, was used to test for changes in sCr, that is, to evaluate whether contrast-induced acute kidney injury (CI-AKI) had occurred. CI-AKI is defined as an increase in sCr of ≥26 μmol/l in the 48–72 h following CM administration or a sCr increase of ≥1.5–1.9 times baseline.^17^

## Results

**Figure 1. (a-d):**
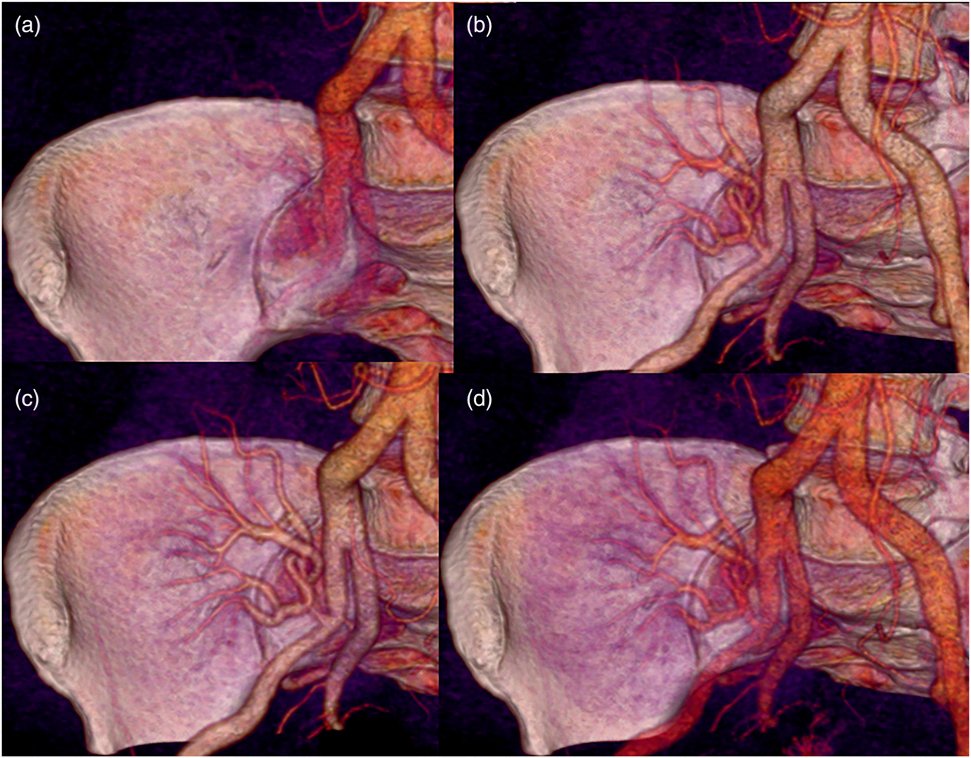
3D volume rendered (VR) images of the arterial anatomy adjacent to the transplant kidney. The images are reconstructed from 4 different P-CTA series, 1.5 time resolution per series.

**Figure 2.**
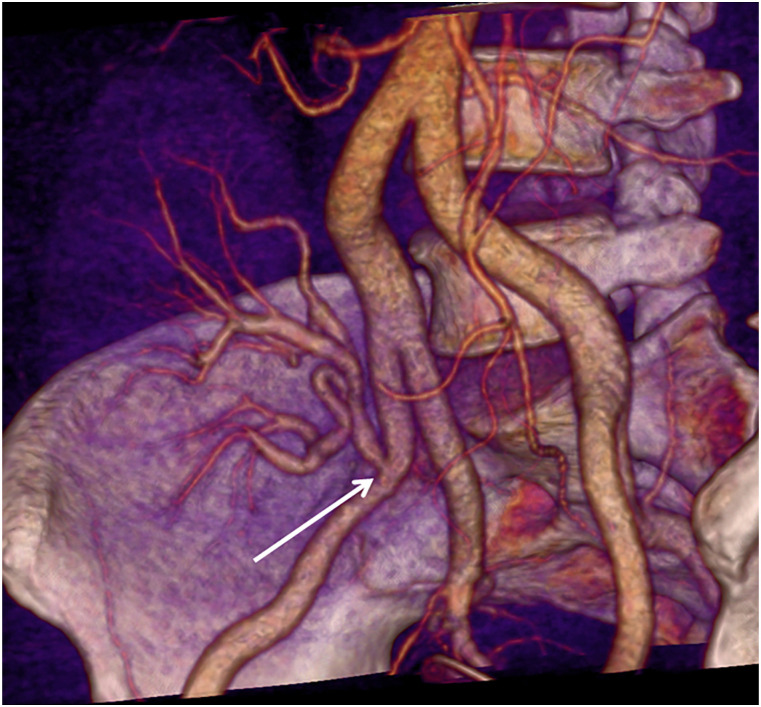
Stenosis of the renal artery at the anastomosis (arrow).

**Figure 3.**
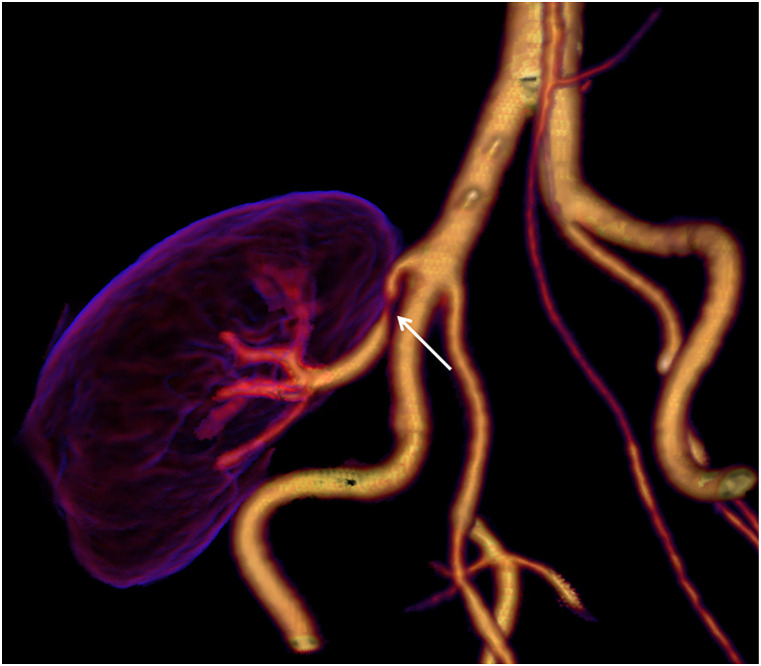
Significant stenosis of the renal artery (arrow). After CT examination, the patient was referred for acute percutaneous transluminal angioplasty (PTA), confirming the renal CTA finding.

**Figure 4.**
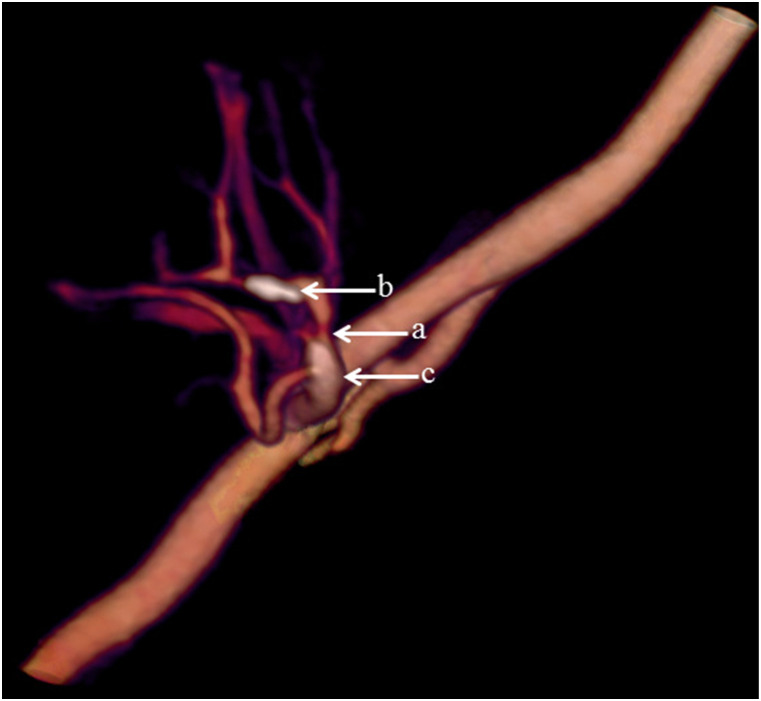
Significant stenosis (arrow a) and calcifications (arrow b, c) of the renal artery.

**Figure 5.**
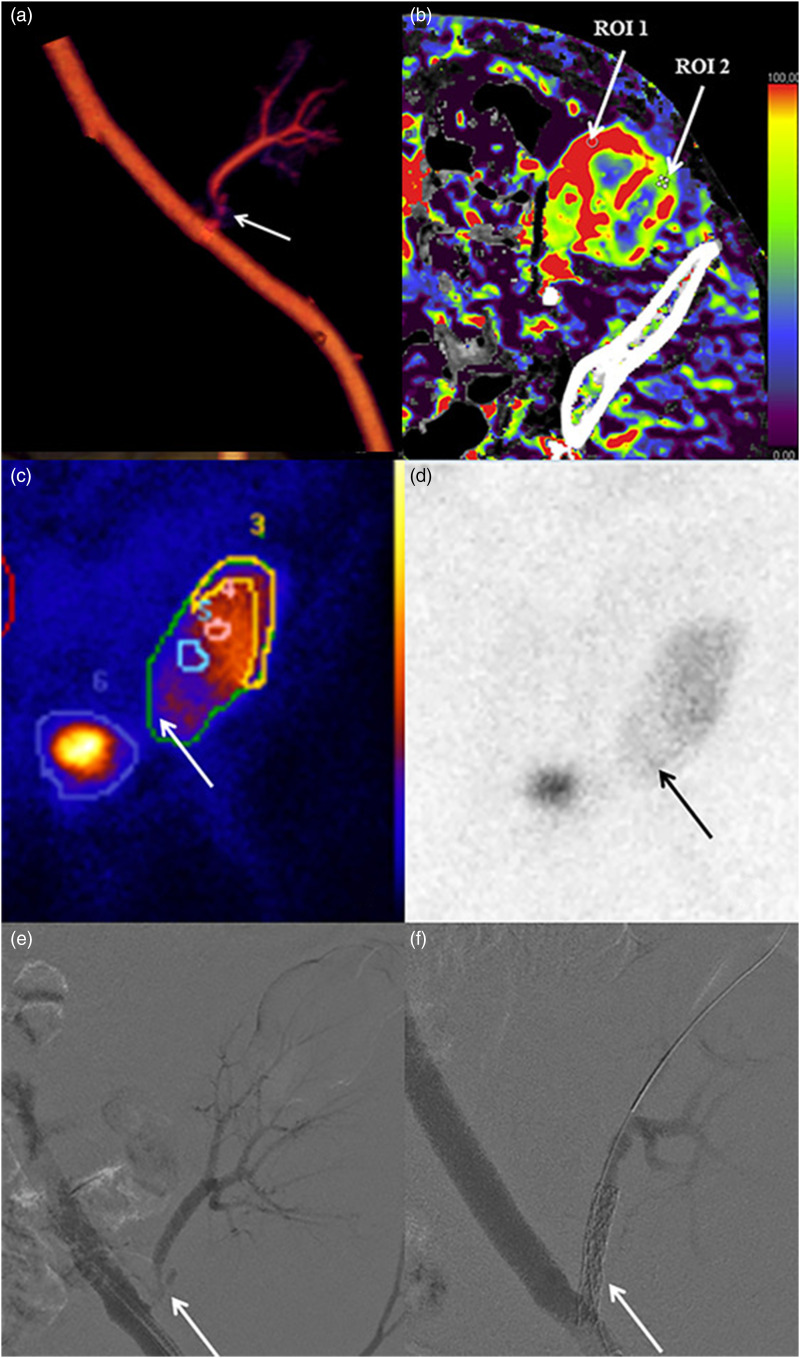
**(a–f)**. Volume rendered CT image **(a)** showing stenosis of the renal artery. Color-coded CT perfusion **(b)** using the same data set acquired from the P-CTA showing decreased perfusion of the caudal renal cortex (ROI 2), perfusion defect confirmed by scintigraphy **(c, d)**. Patient was referred for digital subtraction angiography (DSA) and angioplasty **(e, f)**.

Average body weight was 76 kg (range 55–97 kg). The renal artery of the transplanted kidney was successfully imaged with image quality graded as good or excellent in all 13 patients by both reviewers (Table 2). All arterial anastomoses were done end-to-side to the external iliac artery. For three patients, a donor aortic cuff was used, and for the other 10 patients, the anastomosis was done without cuff. Intravenous CM inflow could be demonstrated with time-resolved images in all patients (Figure 1(a)–(d)), and pathological changes were demonstrated in 10 patients; nine with renal artery stenosis (Figures 2–5), of which one was due to renal atherosclerosis, and one with a kinked renal artery. The diagnosis was verified by DSA in eight patients, of whom six received balloon dilatation and one received a stent. In one patient, a normal artery was verified. In two patients, with suspected stenosis at P-CTA, the kidney function was stabilized and no DSA was performed. In the remaining three patients, no further imaging or intervention was done.

**Table 2.** Attenuation in iliac and transplant kidney artery (HU).

Image quality assessment				
Pat. No	Iliac artery	Transplant kidney artery	Reader 1	Reader 2
1	384	337	5	5
2	160	141	4	4
3	154	140	5	5
4	234	215	5	5
5	318	257	5	5
6	263	200	5	5
7	212	201	4	4
8	359	235	5	5
9	260	293	5	4
10	228	222	5	4
11	264	290	5	4
12	230	200	5	5
13	348	584	5	5

As a by-product of the P-CTA, it was also possible to demonstrate whether the vessel stenosis was accompanied by a decreased parenchymal perfusion [Figure 5(b)].

**Figure 6.**
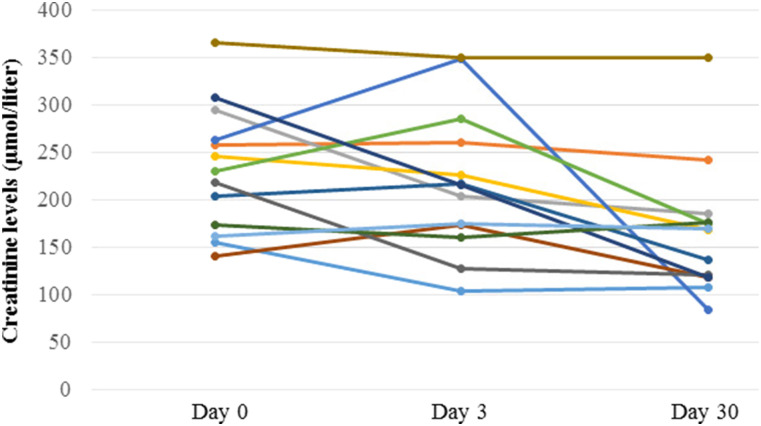
Creatinine levels (μmol/liter) in the 13 patients before, after 3 and 30 days after time-resolved renal CTA.

A transient sCr of >25% was observed in two patients 3 days after the examination. In both patients, the P-CTA showed severe stenosis of the renal artery, which was successfully treated with angioplasty (balloon dilatation and stenting). When analyzing the whole group of patients, there was no significant change in average sCr 3 days after the examination, 219 ± 76 μmol/l, compared with before, 232 ± 66 μmol/l. At 30-day follow-up, sCr had significantly decreased (i.e., normalized) to 165 ± 69 μmol/l (*P* < .01) (Figure 6) (Table 1). The attenuation of the iliac artery ranged between 154 and 384 Hounsfield units (HU, average 263 HU) and of the renal artery between 141 and 584 HU (average 255 HU, Table 2).

## Discussion

The renal artery was successfully visualized by P-CTA in all 13 kidney transplant patients. The image quality was rated as good or excellent in all patients. Six out of eight diagnosed significant stenosis, and two arteries without significant stenosis, were verified at subsequent DSA. In two patients, a stenosis was suspected, but kidney function was regained before DSA. These might constitute false positive cases.

**Figure 7.**
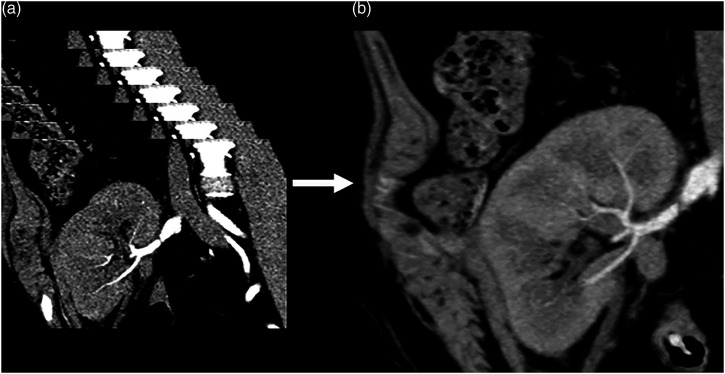
**(a, b)**. Low resolution CT images reconstructed from several time-points **(a)**. By merging data from several time-points, a time-resolved merged data set with high image quality can be reconstructed **(b)**.

When a small amount of CM is injected, as in our study, the CM bolus duration will be very short, which means an increased risk of scanning at the wrong time-point. Through repeated scanning and the use of the same software as that used for reconstruction and analysis of CT-perfusion studies, it was possible to obtain approximately 2000 low-resolution images at five to seven time-points, which eliminates the risk of scanning at the wrong time-point [Figures 7(a) and (b)].

Volume rendering technique (VRT) enabled the imaging of the vascularity of the kidney to be demonstrated with a so-called 4D-movie [Figure 1(a)–(d)]. Such a movie makes it easier for the surgeon and nephrologist to comprehend the anatomy and pathological changes. The data set can also be rotated freely to visualize the anatomy and pathology in the most optimal projection (Figures 2–4). The time-resolved data can also be merged into a single data set of grey-scale images or 3D VRT images with high image quality [Figure 7(b)]. This procedure compensates for the poor image quality of the time-resolved images caused by the low tube current used (70 mAs compared to approximately 150 mAs for standard images). In this study, the resulting image quality was rated good or excellent by the two radiologists. Moreover, scan data can be loaded into the perfusion software to visualize renal perfusion [Figure 5(b)].

Several other reference tests are available to evaluate the renal arteries. The least non-invasive and simplest for the patient is DUS.^18^ In most patients with renal transplants, the kidney is easily imaged as it is located rather superficially and not affected by patient habitus and bowel gas. However, DUS is also user-dependent, and the lack of images that can easily be interpreted by the clinician makes alternative imaging and diagnostic techniques valuable. Gadolinium-enhanced MRA is an appealing alternative,^8,9^ but as mentioned previously, the risk of NSF in patients with severe renal failure and scanner availability limit its use. Non-contrast MRA has shown promising results,^19,20^ but the technique is not yet widely available and there is a risk of occasional false positive results with overestimation of the severity of vascular stenosis.^21^ Standard CTA is not an alternative due to the high CM dose needed, almost 40 g of iodine.^22,23^ A technique to combine CTA with invasive placement of a micro-catheter in the supplying artery has successfully reduced the dose to 5–20 mL,^24^ but this is an invasive technique and requires the interaction of an interventionist. A one-stop-shop alternative is to perform DSA without prior imaging. This would make it possible to diagnose and treat any stenosis at the same time. However, availability, high cost, radiation, and contrast media dose, combined with the invasiveness of the procedure, are limiting factors. P-CTA has the great advantage of using only 8–12 g of iodine, which is less than both DSA, typically 18–33 g of iodine,^25^ and standard CTA.

The risk of causing contrast-induced AKI (CI-AKI) is increased in patients with deteriorating renal function. Some authors argue that the risk of CI-AKI is greatly exaggerated,^26,27^ whereas others argue that there is a considerable risk of CI-AKI in certain patient groups.^17,28^ However, in clinical practice, it is considered that the risk of kidney damage increases with the dose of injected CM, which is especially important to consider in kidney transplant patients.^29,30^ When developing the technique of P-CTA for TRAS, the dosage of CM was based on our previous experience from perfusion CT of the liver using 20 gI (50 mL). As there was no intention to evaluate renal parenchyma enhancement, it was considered that 12 g (30 mL) would suffice. After scanning the first five patients, it became clear that the dose could be further reduced depending on patient habitus. Still, despite the low CM dose, two patients had a serum creatinine increase of >26 μmol/l during the first 3 days. Both patients were diagnosed with severe stenosis of the renal artery [Figure 5(a)–(f)], providing a plausible alternative cause of the kidney injury. Also, one of the patients had no diuresis on the day prior to CTA. Moreover, in all 13 patients, the sCr was significantly lower >30 days after the examination than before, indicating that the CM given at P-CTA did not induce any long-standing kidney damage. However, the retrospective design of the study and the small number of cases make any conclusions on renal function after low contrast dose P-CTA merely speculative.

This study has several limitations. A direct comparison with DSA or MRA to verify the findings in all patients would have been desirable. However, performing DSA in patients with normal findings or stabilized renal function was considered unethical. Furthermore, the reason for developing the low contrast media technique was due to the low availability of MRA. There is also a risk of false positive stenosis at MRA. A possible reason for misinterpretation might be the strong curvature at the anastomosis to the iliac artery that is often observed. Unfortunately, this is also where stenosis most often develops. However, the lack of comparative DSA or MRA means that definite conclusions about the ability of P-CTA to determine if stenosis is present or absent cannot be drawn regarding the remaining five patients.

As this study describes a new technique in a vulnerable group of patients, the patients referred had a high risk of TRAS. This might have biased the reviewers’ evaluations.

It might be possible to use even lower doses of iodine than those used in this study. However, as only two patients out of 13 had possible CI-AKI, it was considered that the risk of non-diagnostic imaging by reducing the dose further would exceed the eventual benefit. The radiation dose during P-CTA was below <9 mSv, equivalent to a standard parenchymal phase liver CT scan. However, the local organ/tissue dose over the transplant kidney and its surroundings was higher due to the repeated scanning over the 15 cm scan length. Regarding availability, the technique of P-CTA should be increasingly available with the greater spread of modern multi-detector CT (MDCT) scanners, provided that it is accepted among radiologists and clinicians. In conclusion, perfusion technique CTA enables imaging of the transplanted kidney by using only a low dose of iodine, reducing the risk of CI-AKI.
